# Alcohol-based hand rubs can fulfil efficacy requirements of EN 1500 in 15 seconds

**DOI:** 10.3205/dgkh000496

**Published:** 2024-08-21

**Authors:** Erika Mönch, Astrid Bolten, Heide Niesalla, Christoph Senges

**Affiliations:** 1 BODE Chemie GmbH, a company of the HARTMANN GROUP, Hamburg, Germany; 2 HARTMANN SCIENCE CENTER, BODE Chemie GmbH, a company of the HARTMANN GROUP, Hamburg, Germany

**Keywords:** hand hygiene, alcohol-based hand rubs, EN 1500, hand antiseptics, efficacy requirements

## Abstract

**Aim:**

Correct hand hygiene is widely regarded as an important measure to prevent healthcare-associated infections. Guidelines on how to perform hand antisepsis are often inspired by laboratory tests that focus on reproducibility rather than ease of use. These cumbersome recommendations can become barriers to hand hygiene, as optimal user acceptance requires a small rub volume and a short application time with an intuitive rubbing technique. Here we modified the EN 1500 to test the efficacy of hand rubs under more user-friendly conditions, using a highly intuitive rubbing technique in 15 seconds.

**Methods:**

The efficacy of an ethanolic and a propanolic hand rub in inactivating *E. coli* on the hands of volunteers was tested according to EN 1500 with modifications in rubbing technique and time. Pre-tests were conducted to find a suitable volume for “responsible application”, a procedure without clearly defined steps. Finally, 20 volunteers applied both rubs for 15 seconds using 3 mL and “responsible application” and 5 mL using the WHO 6-step technique.

**Results:**

Both hand rubs, ethanolic and propanolic, were non-inferior to an unmodified EN 1500 reference for both application methods, 3 mL with “responsible application” and 5 mL with the WHO 6-step method.

**Conclusion:**

Reducing the complexity of hand rub application can have a positive impact on hand hygiene adherence. With our results showing that antimicrobial efficacy comparable to an unmodified EN 1500 can be achieved in 15 seconds using an intuitive rubbing technique, further barriers to more user-friendly hand rub application have been removed.

## Zusammenfassung

**Zielsetzung:**

Korrekte Händehygiene ist als wichtige Maßnahme zur Prävention nosokomialer Infektionen anerkannt. Leitlinien für die korrekte Durchführung der Händedesinfektion stützen sich häufig auf Labortests, bei denen der Schwerpunkt eher auf der Reproduzierbarkeit als auf der Einfachheit der Anwendung liegt. Diese umständlichen Empfehlungen können zu einem Hindernis für die Händehygiene werden, da für eine optimale Anwenderakzeptanz ein geringes Desinfektionsmittel-Volumen und eine kurze Anwendungszeit mit einer intuitiven Einreibetechnik erforderlich sind. Vor diesem Hintergrund haben wir die Wirksamkeit alkoholischer Händedesinfektionsmittel unter anwenderfreundlichen Bedingungen mit einer intuitiven Einreibetechnik über 15 Sekunden anhand einer modifizierten EN 1500 untersucht.

**Methoden:**

Die Wirksamkeit eines ethanolischen und eines propanolischen Hände-Desinfektionsmittels gegen *E. coli* auf den Händen wurde nach der EN 1500 mit modifizierter Einreibetechnik und Einreibezeit geprüft. In Vorversuchen wurde ein geeignetes Volumen für die „eigenverantwortliche Einreibemethode“, ein Verfahren ohne klar definierte Schritte, ermittelt. Schließlich trugen 20 Testpersonen beide Desinfektionsmittel 15 Sekunden lang auf, und zwar mit 3 ml und der „eigenverantwortlichen Einreibemethode“ sowie mit 5 ml und der 6-Schritt-Technik der WHO.

**Ergebnisse:**

Beide Desinfektionsmittel, ethanolisch und propanolisch, waren bei beiden Applikationsmethoden, 3 ml mit der „eigenverantwortlichen Einreibemethode“ als auch 5 ml mit der 6-Schritt-Technik der WHO, einer unmodifizierten Referenz der EN 1500 nicht unterlegen.

**Schlussfolgerung:**

Die Vereinfachung der Anwendung von Hände-Desinfektionsmitteln kann sich positiv auf die Händehygiene-Adhärenz auswirken. Unsere Ergebnisse zeigen, dass mit einer intuitiven Einreibetechnik innerhalb von 15 Sekunden eine antimikrobielle Wirksamkeit erreicht werden kann, die mit der unveränderten EN 1500 vergleichbar ist.

## Introduction

Hand hygiene is considered the most important hygiene measure to prevent healthcare-associated infections [1], [2]. For hygienic hand antisepsis, alcohol-based hand rubs (ABHRs) are used to inactivate the transient skin flora. Three requirements must be met to ensure patient and staff safety: I) an effective hand rub must be used, II) the rub must be applied correctly with sufficient volume, rubbing time and technique, III) the rub must be applied at the right indications. Compliance with all three requirements is often referred to as hand hygiene adherence. Whilst the efficacy of a professional ABHR is systematically verified through standardised testing, for example based on the European Standard EN 1500:2013 [3] (herein referred to as EN 1500), the application should be as simple and quick as possible to reduce any negative impact on usage at the right indications and to ensure patient safety. 

To meet the efficacy requirements of the EN 1500, a tested hand rub must be reproducibly statistically non-inferior to the reference method in inactivating *E. coli* on heavily contaminated hands. Application guidelines for clinical practice, such as strict rubbing techniques for 30 seconds, are often influenced by test methods such as the EN 1500, as these guarantee effectiveness. While it is important to test the efficacy of antiseptics in a standardised and comparable way in the laboratory, the requirements in clinical practice are different. Since staff shortages and high workloads are common problems faced by healthcare workers (HCWs) today, the use of ABHRs in practice often deviates from the recommended procedures. Therefore, complex recommendations to apply at least 3 mL of ABHR by rubbing for 30 seconds following a specific technique, such as the World Health Organization (WHO) 6-step rubbing method, are seen as a barrier and lead to reduced hand hygiene adherence [4], [5], [6]. Alternative rubbing techniques that do not sacrifice safety for simplicity, such as “responsible application”, have been shown to provide comparable skin coverage and may be superior in clinical practice. As HCWs also struggle with the time requirements of hand hygiene [5], reducing application times from 30 to 15 seconds is associated with increased frequency of ABHR usage and hand hygiene compliance [4], [5].

With recommendations to reduce rubbing times to 15 seconds becoming more common in clinical practice, we investigated whether ethanolic and propanolic hand rubs could meet the unmodified efficacy requirements of EN 1500, the baseline for professional hand rubs, in 15 seconds instead of 30 seconds using “responsible application” and/or the standard EN 1500 rubbing procedure.

## Methods

### Study design 

This was a laboratory study with 10 (pre-tests) and 20 (efficacy tests) volunteers, respectively, to assess whether ethanolic and propanolic hand rubs could meet the unmodified efficacy requirements of EN 1500 in 15 seconds. Efficacy tests according to EN 1500:2013 [3] were performed by an independent laboratory accredited according to EN ISO/IEC 17025. 

### Efficacy tests according to EN 1500

All tests were carried out in accordance with EN 1500:2013 with only minor modifications, the reference procedure was never modified [3]. In brief, in a cross-over design, volunteers’ hands were contaminated with *E. coli* K12 NCTC 10538, after which either an ABHR or the reference (60% v/v isopropanol) was applied before the hands were rubbed in culture medium with a neutraliser, which was then poured into plates to count colony forming units (CFU). The reference procedure was always performed as described in the EN 1500 by applying 2x3 mL of 60% v/v isopropanol for 2x30 seconds using the standard rubbing procedure according to Annex A of EN 1500 [3], which is based on the WHO 6-step method. In the following, this rubbing method is called WHO 6-step method. Rubbing times of 15 seconds were tested with an ethanolic hand rub (85% w/w ethanol) and a propanolic hand rub (45% w/w propan-2-ol, 30% w/w propan-1-ol) (both BODE Chemie GmbH, a company of the HARTMANN GROUP, Hamburg, Germany). Allowing only 2 seconds per step, the WHO 6-step rubbing method was tested with 5 mL of ABHR. The WHO 6-step method starts with rubbing the palm and dorsum of the hand, then focuses on the fingertips, which are the most contaminated, for the last three steps [3]. Starting from 3 mL, which is often considered the minimum in EN 1500, a pre-test was performed with 10 volunteers to determine the most appropriate volume (3 mL vs. 4 mL vs. 5 mL) for “responsible application” according to Kampf et al. [7]. Subsequently, 3 mL was used for the EN 1500 efficacy tests with the required 20 volunteers. For the “responsible application”, no specific order or steps are provided, only that the hand should be covered in the given time and that care should be taken to wet the fingertips and thumbs, which are particularly important [7]. The EN 1500 acceptance criteria were met in all efficacy tests with 20 volunteers.

### Data evaluation and statistical analysis

Data analysis was performed as described in EN 1500 [3]. Results were analysed by calculating mean values and standard deviations. Hodges-Lehmann confidence limits were calculated to demonstrate non-inferiority to the reference procedure. In accordance with EN 1500, a non-inferiority limit of 0.6 was used, which should not be exceeded.

## Results

### Pre-tests to define the volume for “responsible application”

Prior to EN 1500 efficacy testing, pre-tests were conducted with 10 volunteers to determine the most appropriate volume for “responsible application”. In terms of antimicrobial efficacy, as demonstrated by log10 reduction, all three volumes for both rubs were comparably effective to the reference (Table 1 (Tab. 1)). Therefore, to evaluate the most convenient method for clinical practice, the lowest volume (3 mL) was chosen for the EN 1500 with 20 volunteers.

### EN 1500 testing of an ethanolic and a propanolic hand rub in 15 seconds

**Figure 1:**
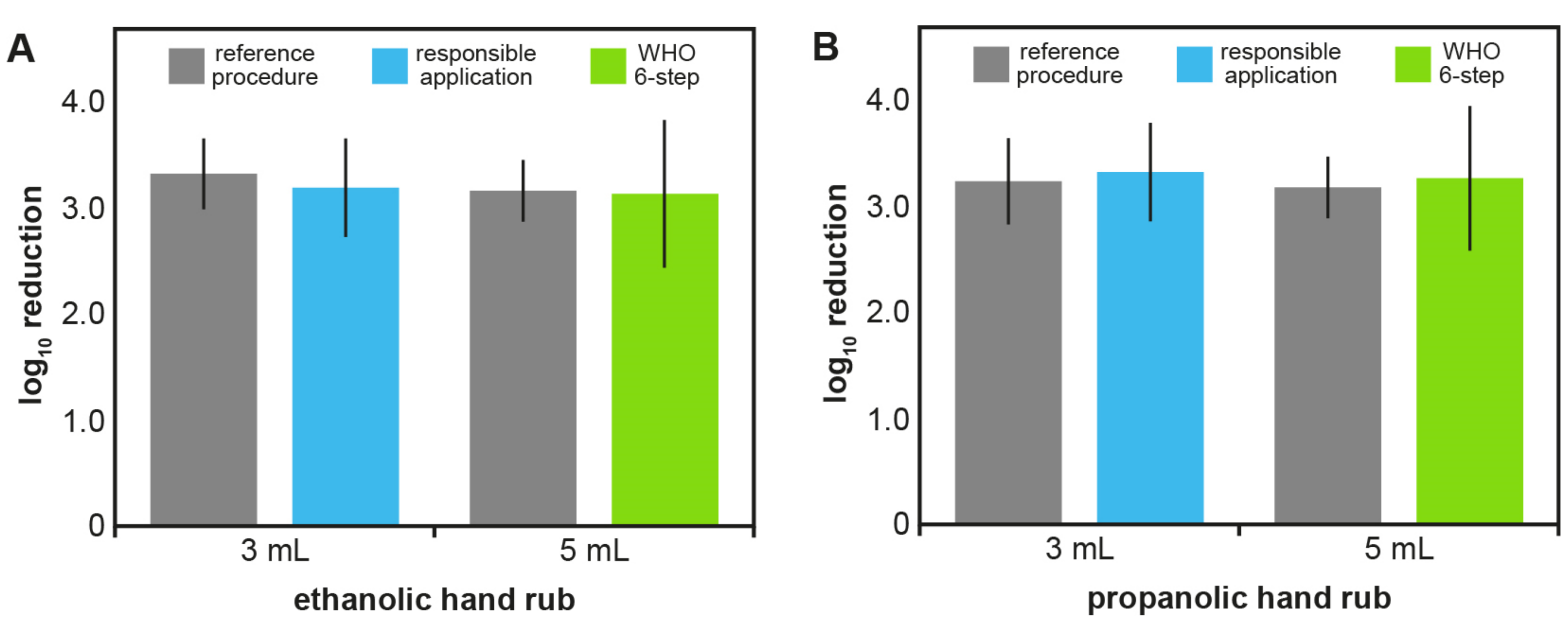
Log10 reductions in the EN 1500 test with 15 seconds of rubbing time. Shown are means (columns) and standard deviations (error bars) of log10 reductions in EN 1500 tests with 20 volunteers. Ethanolic (A) and propanolic hand rubs (B) were tested using 3 mL for the “responsible application” (blue) and 5 mL for the WHO 6-step method (green) for 15 seconds. The reference procedure (grey) was not modified from EN 1500 and used 2x3 mL of 60% isopropanol for 2x30 seconds.

Ethanolic and propanolic hand rubs with 15 seconds of rubbing time were non-inferior to the reference when 3 mL was used with “responsible application” or 5 mL with the WHO 6-step method. While the volume for “responsible application” was determined in the pre-test, 5 mL of hand rub were used for the WHO 6-step method to ensure maximum coverage of the hand. Both hand rubs tested were as effective in reducing the *E*. coli contamination as the standard reference procedure and therefore met the requirements of the EN 1500 after 15 seconds for both “responsible application” and the WHO 6-step method (Table 2 (Tab. 2) and Figure 1 (Fig. 1)). Mean log10 reductions by the ethanolic hand rub were 3.20±0.47 for “responsible application” and 3.16±0.70 for the WHO 6-step method, whereas the reference procedure achieved reductions of 3.33±0.26 and 3.17±0.28, respectively (Table 2 (Tab. 2) and Figure 1 A (Fig. 1)). Similar results were obtained for the propanolic hand rub, with mean log10 reductions of 3.33±0.46 (“responsible application”) and 3.27±0.69 (WHO 6-step method), whereas the reference procedure resulted in reductions of 3.25±0.40 and 3.17±0.28, respectively (Table 2 (Tab. 2) and Figure 1B (Fig. 1)). Interestingly, in both cases the standard deviations were larger when a volume of 5 mL was used. For both hand rubs and both application techniques, the Hodges-Lehmann confidence limits were less than 0.6, confirming non-inferiority in 15 seconds.

## Discussion

Since healthcare-associated infections are mainly transmitted via hands, hand hygiene is the most important measure for their prevention [1], [2]. Therefore, firstly, a hand rub like an alcohol-based hand rub (ABHR) must be effective to ensure patient and staff safety. Secondly, an ABHR needs to be easy to use to ensure correct application at the right indication. While standardised processes such as the EN 1500 are undoubtedly required for comparable testing, the user-friendliness of the rubbing methods in everyday clinical practice is not sufficiently considered [6]. For high user acceptance a small ABHR volume and a short application time with a highly intuitive rubbing technique would be optimal [6]. As high alcohol (85% w/w) ethanolic hand rubs, such as the one tested here, are known to consistently meet EN 1500 within 30 seconds of rubbing, there may be potential for simplified application [8], [9].

In clinical practice, application times are usually shorter than the often recommended 30 seconds, with physicians, for example, using 8.5 seconds on average [10]. Interestingly, many HCWs tend to use rub volumes that give them “acceptable” drying times, which appear to be 15 to 20 seconds [11]. But shorter application times are not necessarily disadvantageous: under controlled conditions, hand coverage in 15 seconds is comparable to 30 seconds especially with trained subjects [12], [13], and Pires et al. showed that reduction of bacterial CFUs after 10 to 20 seconds of hand rubbing was not significantly different from that after 30 seconds or more [13]. In addition, a study on a neonatal intensive care unit showed no negative effects in terms of antiseptic efficacy but an increase in hand hygiene adherence [5]. This is also reflected in the current recommendations of the Society for Healthcare and Epidemiology of America (SHEA), which considers 15 seconds to be the minimum to achieve full hand coverage [14], [15]. As it takes an average of 42 to 67 seconds to dry 3 mL of ABHR, and 30 seconds is therefore not sufficient for complete drying, it appears that rubbing is usually stopped before the hands are dry, even in clinical practice [16], [17]. However, even without rubbing, antiseptics continually inactivate the skin flora until the skin has dried.

Interestingly, when evaluating the volumes used, we observed larger standard deviations of the log10 reduction rates when using the larger ABHR volume (5 mL) compared to 3 mL. This is in line with the work of Voniatis et al., who showed that too large volumes can reduce the quality of hand antisepsis through increased dripping [18], while 2.25 mL can be considered to be the minimum for adequate hand coverage [11], [18]. In addition to rubbing times being too long, strict rubbing techniques such as the WHO 6-step technique are often perceived as too complex, and simpler methods may lead to an additional increase in hand hygiene adherence [6], [7]. In addition to clinical studies showing that 15 seconds of rubbing can be as good as or better than 30 seconds, our results show that with ethanolic and propanolic hand rubs, microbial safety equivalent to EN 1500 can be achieved within 15 seconds using the highly intuitive “responsible application” technique [4], [5]. 

### Limitations

Whilst the aim of this paper was to bridge the gap between standardized laboratory tests and clinical practice, all our investigations were based on standardized tests. At 3.17 to 3.33 log CFU reduction for the reference procedures, our results are at the lower end of a recently published interlaboratory comparison [8]. Given that some guidelines recommend rubbing hands until dry, drying time is an important factor that we have not addressed [13]. Drying times are influenced by many factors, including the hands of the HCW and the formulation of the ABHR used. Here we have only tested one ethanolic and one propanolic ABHR and cannot assess to what extent these data apply to other ABHRs. In addition, following the EN 1500 protocol, we have neither recorded hand sizes, nor did we adjust volumes or evaluate data based on size information. A recent inter-laboratory ring trial showed that the size of the subject’s hand did not significantly affect the results of the EN 1500 [8]. Given that it takes 42 to 67 seconds to dry 3 mL of an ABHR, it appears to be common practice for HCWs to continue working with alcohol-wet hands [17]. We have not investigated whether this is worsened or improved by alternative rubbing techniques or the use of 5 mL ABHR, nor did we evaluate appropriate patient care tasks that HCWs could perform to bridge drying times.

## Conclusions

Previous work has indicated that intuitive rubbing techniques and application times of less than 30 seconds may increase hand hygiene adherence and do not necessarily decrease safety in practice. With our work showing that ABHRs, both ethanolic and propanolic, can ensure microbial safety with “responsible application” in 15 seconds, further barriers to a more user-friendly ABHR application have been removed. Future research may lead to further simplification while maintaining microbiological safety, helping to further optimise the process for practical use and increase user acceptance, thereby improving hand hygiene adherence rates. 

## Notes

### Competing interests

EM, AB, HN, and CS are employees of BODE Chemie GmbH, a company of the HARTMANN GROUP (Hamburg, Germany), the manufacturer of the tested hand rubs.

### Authors’ ORCIDs

- Heide Niesalla: 0000-0002-5992-5820
- Christoph Senges: 0000-0002-8069-8730

### Ethical approval 

The experiments in this study were conducted by an independent EN ISO/IEC 17025 accredited laboratory, acting as a contract research agency, following the EN 1500:2013 protocol with two market-approved alcohol-based hand rubs. Ethics board approval was not required based on the classification of *Escherichia coli* K12 (NCTC 10538) as a Risk Group 1 non-pathogenic organism by the German Safety Ordinance on Gene Technology. All volunteers gave written informed consent, and no personal data was shared with the study sponsors.

### Funding 

This work and the experiments performed by an independent laboratory accredited according to EN ISO/IEC 17025 were financed by BODE Chemie GmbH, a company of the HARTMANN GROUP (Hamburg, Germany).

### Acknowledgments 

Medical writing assistance was provided by Dr. Julia Dittmann (Dittmann Medical Writing, Hamburg, Germany) and financed by BODE Chemie GmbH, a company of the HARTMANN GROUP (Hamburg, Germany). 

## Figures and Tables

**Table 1:**
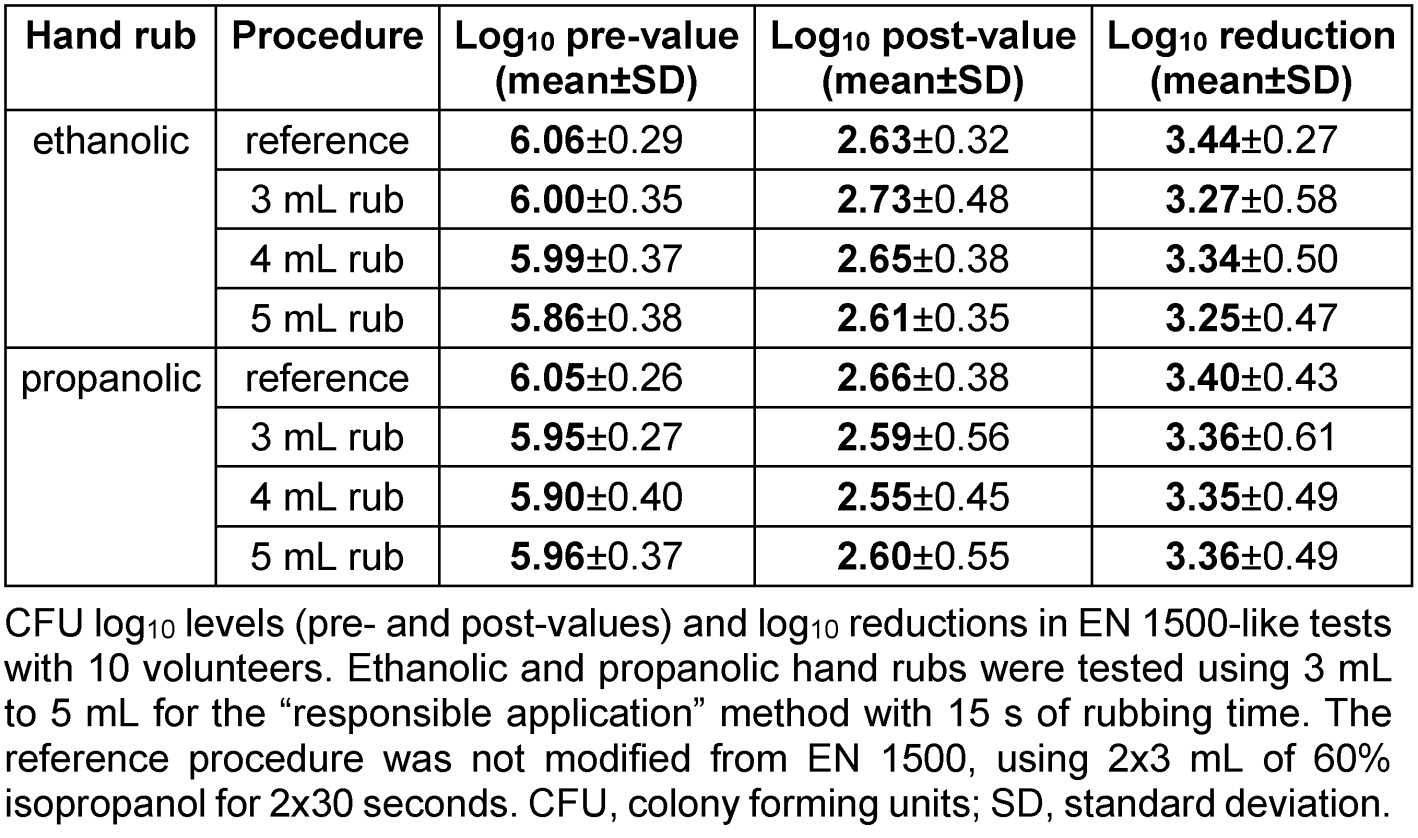
Comparison of hand rub volumes in EN 1500 using “responsible application” in 15 seconds.

**Table 2:**
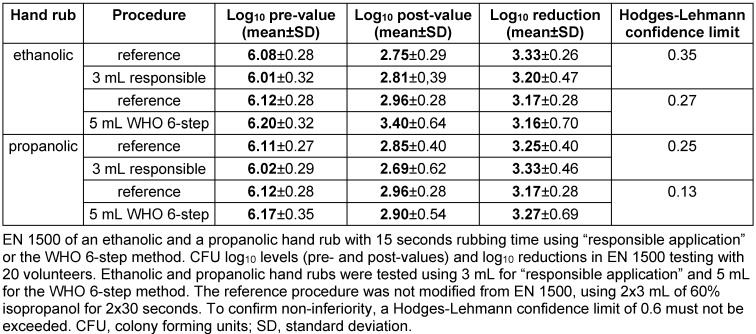
EN 1500 in 15 seconds using two hand rubs and two rubbing techniques.

## References

[1] Hauer T, Dettenkofer M (2014). Epidemiologie und Prävention von nosokomialen Infektionen. Pädiatrie.

[2] Lotfinejad N, Peters A, Tartari E, Fankhauser-Rodriguez C, Pires D, Pittet D (2021). Hand hygiene in health care: 20 years of ongoing advances and perspectives. Lancet Infect Dis.

[3] (2013). DIN EN 1500-2013: Chemical disinfectants and antiseptics - Hygienic handrub - Test method and requirements (phase 2/step 2).

[4] Harnoss JC, Dancer SJ, Kaden CF, Baguhl R, Kohlmann T, Papke R, Zygmunt M, Assadian O, Suchomel M, Pittet D, Kramer A (2020). Hand antisepsis without decreasing efficacy by shortening the rub-in time of alcohol-based handrubs to 15 seconds. J Hosp Infect.

[5] Kramer A, Pittet D, Klasinc R, Krebs S, Koburger T, Fusch C, Assadian O (2017). Shortening the Application Time of Alcohol-Based Hand Rubs to 15 Seconds May Improve the Frequency of Hand Antisepsis Actions in a Neonatal Intensive Care Unit. Infect Control Hosp Epidemiol.

[6] von Lengerke T, Schulz-Stübner S, Chaberny IF, Lutze B (2016). Psychologie der Händehygiene-Compliance: Von der Motivation zum Verhalten. Krankenhaushygiene Up2date.

[7] Kampf G, Reichel M, Feil Y, Eggerstedt S, Kaulfers PM (2008). Influence of rub-in technique on required application time and hand coverage in hygienic hand disinfection. BMC Infect Dis.

[8] Suchomel M, Kampf G, Gebel J, Droop F, Christiansen B, Roesch KM (2024). How reliable are test results from 17 laboratories on the basis of EN 1500 for a hand rub based on 80% (w/w)? J Hosp Infect.

[9] Macinga DR, Shumaker DJ, Werner HP, Edmonds SL, Leslie RA, Parker AE, Arbogast JW (2014). The relative influences of product volume, delivery format and alcohol concentration on dry-time and efficacy of alcohol-based hand rubs. BMC Infect Dis.

[10] Stahmeyer JT, Lutze B, von Lengerke T, Chaberny IF, Krauth C (2017). Hand hygiene in intensive care units: a matter of time? J Hosp Infect.

[11] Kenters N, Eikelenboom-Boskamp A, Hines J, McGeer A, Huijskens EGW, Voss A (2020). Product dose considerations for real-world hand sanitiser efficacy. Am J Infect Control.

[12] Paula H, Becker R, Assadian O, Heidecke CD, Kramer A (2018). Wettability of hands during 15-second and 30-second handrub time intervals: A prospective, randomized crossover study. Am J Infect Control.

[13] Pires D, Soule H, Bellissimo-Rodrigues F, Gayet-Ageron A, Pittet D (2017). Hand Hygiene With Alcohol-Based Hand Rub: How Long Is Long Enough?. Infect Control Hosp Epidemiol.

[14] Glowicz JB, Landon E, Sickbert-Bennett EE, Aiello AE, deKay K, Hoffmann KK, Maragakis L, Olmsted RN, Polgreen PM, Trexler PA, VanAmringe MA, Wood AR, Yokoe D, Ellingson KD (2023). SHEA/IDSA/APIC Practice Recommendation: Strategies to prevent healthcare-associated infections through hand hygiene: 2022 Update. Infect Control Hosp Epidemiol.

[15] Tartari E, Bellissimo-Rodrigues F, Pires D, Fankhauser C, Lotfinejad N, Saito H, Suchomel M, Kramer A, Allegranzi B, Boyce J, Sax H, Stewardson AJ, Pittet D, ICPIC Alcohol-Based Handrub Task Force (2024). Updates and future directions regarding hand hygiene in the healthcare setting: insights from the 3rd ICPIC alcohol-based handrub (ABHR) task force. Antimicrob Resist Infect Control.

[16] Voniatis C, Bánsághi S, Veres DS, Szerémy P, Jedlovszky-Hajdu A, Szijártó A, Haidegger T (2023). Evidence-based hand hygiene: Liquid or gel handrub, does it matter? Antimicrob Resist Infect Control.

[17] Suchomel M, Leslie RA, Parker AE, Macinga DR (2018). How long is enough? Identification of product dry-time as a primary driver of alcohol-based hand rub efficacy. Antimicrob Resist Infect Control.

[18] Voniatis C, Bánsághi S, Ferencz A, Haidegger T (2021). A large-scale investigation of alcohol-based handrub (ABHR) volume: hand coverage correlations utilizing an innovative quantitative evaluation system. Antimicrob Resist Infect Control.

